# Expression of proteins Heat-Labile (Lt-1) and Heat-Stable (STa) *Escherichia coli *expression of proteins Heat-Labile (Lt-1) and Heat-Stable (STa) *Escherichia coli*

**DOI:** 10.1186/1753-6561-8-S4-P43

**Published:** 2014-10-01

**Authors:** Alessandra Cavalcante, Maria Eliza Caldas dos Santos, Luís André Morais Mariúba, Paulo Afonso Nogueira

**Affiliations:** 1 Instituto Leônidas e Maria Deane, Manaus, Amazonas, Brasil

## Introduction

*Escherichia coli *has a direct relationship with the diarrheagenic diseases, which occur quite in developing countries due to poor sanitation system and public health. It mainly affects children, causing a rate of morbidity and mortality worrying.

This bacterium has a versatility of strains expressing different mechanisms and virulence factors, thus the importance in studying the taxonomy of these cepas. Classified into six categories: enteropathogenic E.coli (EPEC), enterotoxigenic *E. coli *(ETEC), enteroinvasive *E. coli *(EIEC), enterohemorrhagic *E. coli *(EHEC), enteroaggregative *E. coli *(EAEC) and diffusely adhering *E. coli *(DAEC). Among them, ETEC is the most common etiologic agent of infantile diarrhea related and has the characteristic resist peristaltic movements and adhere to the small intestine produce enterotoxin. The enterotoxins are proteins encoded by plasmids and occurring in two forms: one stable toxin (ST) which may be of the type Sta and Stb or and a labile toxin (LT), which also can be of two types LT and LT-1 or LT -2. Strains of ETEC causes diarrhea when adhere to enterocytes of the small intestine, mediated by one or more adhesins called CFA (colonization factor antigens).

The development of antisera and specific antibodies identification of species and subspecies of Enterobacteriaceae prevent the misuse of antibiotics, will enable the taxonomic study and facilitate medical diagnosis.

## Methods

This study samples of enterotoxigenic *E. coli *(ETEC) are used the bank's sample Institute Leônidas and Maria Deane (ILMD-FIOCRUZ). After extraction of genomic DNA samples, was made the standardization of PCR. The result was visualized on 1.5% agarose gel and bands for the purification of LT-1 and Sta genes was used QIAquick Gel Extraction Kit QIAGEN. Then, the preparation of competent cell was made with top 10 for cloning and subcloning, DHα5 pLysS and BL21 for expression. Purified fragments were ligated to the vector pGEM T Easy cloning and transformation was performed. The white colonies obtained after transformation of bacteria with binding were subjected to screening by PCR and sent for sequencing. Plasmids were digested by the restriction enzymes EcoR1 and BamH1 cloning vector and pQE (Invitrogen) was digested using enzymes Sph1 and Pst1. Fragments linked to expression vectors were inserted by transformation into BL21 pLysS plasmid and subjected to extraction. Confirmation of the expression of target proteins was performed by SDS-PAGE and confirmation of gene expression was used Western blot (Invitrogen).

## Results

Confirmation of cloning was performed by PCR with sense and antisense primer pGEM of the genes chosen allowing increasing the size of the fragment. In the subcloning, we used the restriction enzyme EcoR1 digested that the correct gene Sta, however digested LT-1 gene sequences not expected. Due to this gene for the expression of LT will use an expression plasmid (pQE) different.

Protein expression heat-stable Sta using plasmid pRSETC was seen in Western Blot presenting highest expression pellets and size of 4 kDA.

## References

[B1] Nataro JP Kaper JB Diarrheagenic *Escherichia coli. Clin.* Microbiol Rev 1998 11 1 142 201 10.1128/cmr.11.1.142 PMC121379 9457432

[B2] Aguerro ME Reyes L Prado V Orskov I Orskov F Cabello FC Enterotoxigenic *Escherichia coli *in a population of infants with diarrhea in Chile J Clin Microbiol 1985 22 4 576 58 3908470 10.1128/jcm.22.4.576-581.1985 PMC268470

[B3] Sambrook J Fritsch EF Maniatis T Molecular cloning: A laboratory manual 1989 1,2 and 3 2

[B4] Gomes TA Rassi V MacDonald KL Ramos SR Trabulsi SR Vieira MA Guth BE Ivey C Toledo MR Blake PA Enteropathogens associated with acute diarrheal disease in urban infants in São Paulo, Brazil J Infect Dis 1991 164 2 331 337 10.1093/infdis/164.2.331 1856482

